# Manipulating Molecular Self-Assembly Process at the Solid–Liquid Interface Probed by Scanning Tunneling Microscopy

**DOI:** 10.3390/polym15204176

**Published:** 2023-10-20

**Authors:** Zhi Li, Yanan Li, Chengjie Yin

**Affiliations:** School of Chemical Engineering, Anhui University of Science and Technology, Huainan 232001, China; 2022025@aust.edu.cn

**Keywords:** self-assembly, scanning tunneling microscopy, external stimuli, phase transition

## Abstract

The phenomenon of ordered self-assembly on solid substrates is a topic of interest in both fundamental surface science research and its applications in nanotechnology. The regulation and control of two-dimensional (2D) self-assembled supra-molecular structures on surfaces have been realized through applying external stimuli. By utilizing scanning tunneling microscopy (STM), researchers can investigate the detailed phase transition process of self-assembled monolayers (SAMs), providing insight into the interplay between intermolecular weak interactions and substrate–molecule interactions, which govern the formation of molecular self-assembly. This review will discuss the structural transition of self-assembly probed by STM in response to external stimuli and provide state-of-the-art methods such as tip-induced confinement for the alignment of SAM domains and selective chirality. Finally, we discuss the challenges and opportunities in the field of self-assembly and STM.

## 1. Introduction

With the increasing need for downsizing in the electronics industry, the development of molecular electronic devices presents a promising solution to overcome the limitations imposed by silicon-based electronics [1,2]. The conventional silicon electronics has relied on lithographic patterning of polymer resists at progressively smaller lengths to scale down device dimensions. However, this approach has its limitations. In response, numerous fabrication techniques have been explored to create molecular electronic devices, resulting in significant advancements in fabrication, characterization, and application within this field [3,4,5].

One of the hottest topics is to recognize and manipulate the functions and lateral structures of molecules at the nanoscale during two-dimensional (2D) supramolecular self-assembly process [6]. Molecular self-assembly is a spontaneous process [7,8,9], during which molecules are held together into ordered structures via intra- and intermolecular non-covalent interactions such as hydrogen bonding [10,11], van der Waals force [12], π-π stacking [13,14] and electrostatic force [15,16]. In assistance of an atomically flat substrate, 2D self-assembled networks can be stabilized on the surface of the substrate based on the noncovalent interactions between molecules and substrates under thermodynamic control. In order to tune the functions and structures, the ordered 2D patterns can go through a structural and functional transition into another one with external stimuli, such as solvents [17,18,19], thermal treatment [20,21], varying concentrations [22,23], light irradiation [24], electric field [25,26], and guest-molecule-induced co-adsorption and desorption processes [27,28].

Since its invention in 1981, scanning tunneling microscopy (STM) has proven to be a powerful technique for visualizing 2D materials and molecular networks, providing direct proofs for the way of molecular bonding and phase transition at the solid–liquid interface [29]. Owing to this significant contribution, Gerd Binning and Heinrich Rohrer were awarded with the Nobel Prize of Physics in 1986. The principle of STM is simple. When an atomically sharp conductive STM tip is close enough to a conductive substrate, a tunneling current is generated through the tip and the substrate upon applying a sufficient voltage. The tunneling current exponentially changes with the distance between the tip apex and the measured atom on the substrate. Based on the relationship between the tunneling current and the distance, a STM image with atomic resolution can be generated when the tip is scanning over the substrate. Therefore, STM can be very useful to monitor the molecular structure formed during self-assembly, molecular dynamics under thermodynamics and surface chirality. Furthermore, external forces from the STM tip or flow can also be applied to trigger the phase transition process of self-assembled molecular networks.

In this contribution, we focus on the recent progress of controlling the external stimuli to achieve state-of-the-art manipulation of phase transition of 2D molecular networks probed by STM at the solid–liquid interface, including temperature, electric field, photo-irradiation, shear-flow, and tip induction.

## 2. Self-Assembled Structures Induced by Thermal Treatment

As the molecular self-assembly process is governed under thermodynamic control [30,31,32], thermal treatment is a critical factor to manipulate the phase transition process of 2D molecular networks. Moreover, thermal treatment is a widely recognized process that can lead to the creation of an organized adlayer or the development of a new thermodynamically stable structure, or result in the expansion of the domain size [33]. Azzam et al. demonstrated a continuous decrease in the surface coverage of self-assembled monolayers (SAMs) of terphenylethanethiol on Au (111) and dramatic alterations in the substrate morphology caused by the partial desorption of molecules with elevated temperatures [34]. Interestingly, the SAMs of terphenylethanethiol experienced a consecutive α-β-γ-δ phase transitions upon thermal annealing from the room temperature up to 468 K. At the room temperature, a densely packed a α phase with a $\left(2\sqrt{3}\times \sqrt{3}\right)R30{}^{\circ}$ structure was observed on the Au (111) substrate, while it changed to a β phase with a $\left(3\sqrt{3}\times 5\right)$ structure, associated with the appearance of irregular gold islands, upon annealing at 333 K. Further annealing to 373 K promoted the formation of a γ phase with a (2$\sqrt{5}\times 4)R7{}^{\circ}$ structure. A final δ phase (2$\sqrt{5}\times \sqrt{7})R13{}^{\circ}$ appeared upon annealing at the temperature higher than 468 K. The packing density of the molecules continuously decreased with the phase transition at higher temperatures due to the thermal-induced partial desorption of molecules.

**Figure 1:**
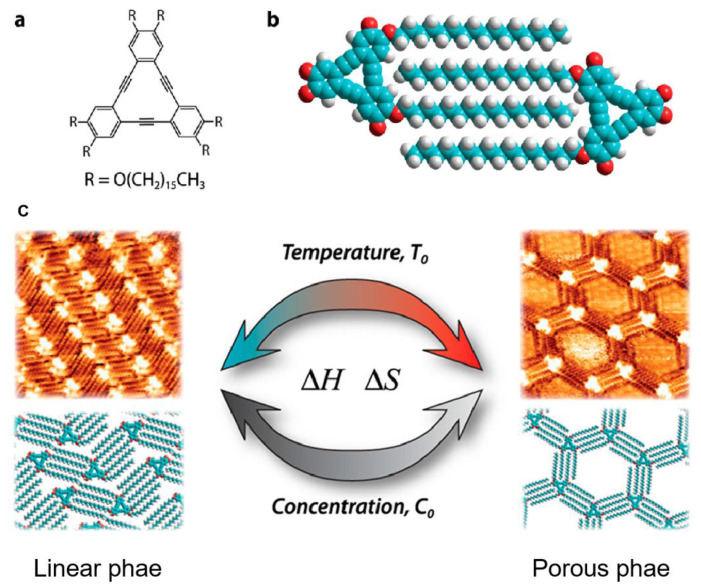
Temperature-induced structure transformation of SAMs of DBA derivative on a graphite substrate. (**a**) The chemical structure of DBA-OC16; (**b**) the molecular model demonstrates the interlocking arrangement of alkoxy chains between neighboring DBA-OC16 molecules; (**c**) the temperature and concentration-induced structure transition between the linear and porous phase of DBA-OC16. Reproduced with permission from ref. [20]. Copyright 2013, American Chemical Society.

Similarly, De Feyter et al. realized the temperature-induced structural phase transition between a closely arranged linear and a loosely packed porous phase of SAMs of alkylated dehydrobenzo [12] annulene (DBA) derivative on a graphite substrate. Figure 1a and b showed the chemical structure of DBA-OC16 and the corresponding molecular model of the interlocking arrangement of alkoxy chains between neighboring DBA-OC16 molecules. Following this way, the DBA-OC16 molecules were expected to form linear and porous phases on a flat substrate. At the room temperature, the DBA molecules were co-adsorbed with solvent molecules (1,2,4-trichlorobenzene) as the guests on a graphite surface and arranged into porous structure [20], Figure 1c. Based on thermal annealing up to 353 K, the solvent molecules were evaporated from the pores and the structure were stabilized as a closely packed linear phase under entropic and enthalpic control, Figure 1c. Furthermore, thermal treatment can also help induce the switch of chirality of SAMs. At the room temperature high concentration of chiral DBA(S) molecules appeared on the graphite surface with the co-existence of 81% clockwise (CW) and 19% counter-clockwise (CCW) porous patterns. However, annealing at 80 °C converted the original phases to a homochiral SAMs of CCW pattern by coalescence of domains through a ripening process, which is because that the guests DBA(S) molecules acting as guests preferentially stayed in CCW pores to form stable host–guest systems [35]. Additionally, thermal annealing can also help conduct the trans–cis transformation of SAMs. Wan et al. investigated the temperature effect on the self-assembly behavior of bis(4,4′-(m,m’-di(dodecyloxy)phenyl)-2,2′-difluoro-1,3,2-dioxaborine) on highly oriented pyrolytic graphite (HOPG) [36]. Firstly, a lamellar structure with trans conformation was observed on the HOPG surface at the room temperature with two dioxaborine (DOB) moieties aligned in a dihedral angle of 180 degree. When the temperature was lifted to 100 °C, the quality of the SAMs was improved with long-range ordering of molecular arrays. Heating at 130 °C triggered the trans–cis transformation and a small region with hexagonal structure of cis isomer of the molecules was observed. When heating at 150 °C, the domain of hexagonal structure of cis isomers expanded and eventually accomplished the complete trans–cis transformation by covering the entire surface. Over the course of the past few decades, thermal treatment was mainly applied to the 2D phase transition of SAMs, which lack real applications in the field of nanotechnology. Recently, 2D covalent organic frameworks (COFs) [37,38] and graphene nanoribbons (GNRs) [39,40,41], have become cutting-edge research fields due to their wide-range applications. Surprisingly, thermal treatment has been proven an effective method for the promotion of on-surface synthesis processes to form large-area 2D COFs and GNRs. For example, Shi et al. developed a three-step annealing process for the synthesis of 2D COF starting from 4-bromo-4″-chloro-5′-(4-chlorophenyl)-1,1′ : 3′,1″-terphenyl (BCCTP) monomer, which has two chloride ends and one bromide end [37]. The BCCTP monomers selectively react with each other at the bromide end to form dimers on the Au (111) substrate at 180 °C. Subsequently, in assistance of Cu, oligomers were formed by annealing at 140 °C for 10 min. Further annealing at 280 °C for 30 min promotes the formation of 2D covalent frameworks. The utilization of a hierarchical process of selective reactivity presents a potentially valuable approach for achieving the thermodynamic product and minimizing kinetic trapping during the synthesis of 2D COFs.

## 3. Self-Assembled Structures Tuned by Photo-Induction

Recently, surface photochemistry has been intensively studied, due to its powerful function for tunning SAMs’ ordering and initiating on-surface polymerization, including photoisomerization [42,43], photopolymerization [44,45] and photocycloaddition [46,47]. The SAM adlayer normally experiences significant structural changes as a result of surface photochemical reactions, primarily due to the substantial structural disparity between the photo-active molecules before and after the photo-induction. Pace et al. observed the light-induced SAM phase transition of azobenzene molecules on the Au (111) substrate with STM images. The transformation of the trans-form of SAM to the cis-form was promoted by 360 nm light irradiation, while the reversed transition was performed by 450 nm light irradiation [48].

**Figure 2:**
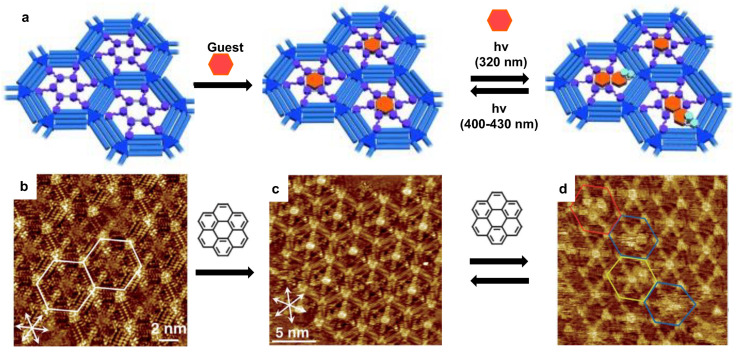
The photo-induced guest adsorption and desorption in a host–guest system. (**a**) The schematic illustration photo-induced guest adsorption and desorption in DBA pores; STM images of (**b**) the trans-DBA pores without coronene guest; (**c**) the trans-DBA/coronene host-guest system; (**d**) the cis-DBA/coronene host-guest system. The white arrows correspond to the main symmetric axes of graphite. The colored hexagons indicate the pores containing four CORs (red), two CORs (yellow), and those with fuzzy images (blue). Reproduced with permission from ref. [49]. Copyright 2013, John Wiley and Sons.

Later, Tahara et al. investigated the photo-induced phase transition of SAMs in a more complex system, involving a photo-induced guest adsorption and desorption process [49]. The schematic illustration clearly demonstrated the photo-induced guest adsorption and desorption process in DBA pores, see Figure 2a. Firstly, the trans-form azobenzene-functionalized DBA self-assembled into a hexagonal porous structure at the 1-octanoicacid/graphite interface, see Figure 2b. After adding the coronene guest molecules into the system, one of each coronene molecule was immobilized in one pore formed by the trans-azobenzene-functionalized DBA, see Figure 2c. Upon light-irradiation at 320 nm, the trans-to-cis transformation occurred with desorption of one or two azobenzene units from the substrate surface, which left sufficient space for the accommodation of additional coronene guest molecules in the pores, see Figure 2d. Reversely, applying a light-irradiation at 400–430 nm initiated the cis-to-trans transformation, which promoted the re-adsorption of the trans-molecules onto the substrate surface and excluded the extra guest molecules. Yokoyama et al. demonstrated the investigation of photo-activated structural switch behavior of a photochromic terthiophene derivative with STM at the solid–liquid interface [50]. No ordered structure was observed for the closed-ring phase of a terthiophene derivative, while it underwent a phase transition to two ordered phases (α-ordering and β-ordering) due to the visible-light-induced ring opening of the terthiophene derivative. When UV light was used for the photo-induction, a third ordered phase (γ-ordering) appeared due to the formation of the annulated isomer. It is also possible to control the conductance of molecular electronics via light-irradiation-induced opening and closing of the ring of the thiophene unit of SAM on Au substrate. A reversible conversion between two distinguishable conductance states can be controlled via photoisomerization of the switches by using alternative irradiation with UV (λ = 313 nm) or visible (λ > 420 nm) light to achieve the on-state and off-state, respectively. This transition process was clearly probed by the STM images, showing the distinct height difference between the one-state (0.4–0.6 nm) and off-state of the molecules (0–0.1 nm) [51]. Recently, photo-induction has been combined with the STM technique to generate a STM junction for building ultrafast photon-induced tunneling microscopy [52]. Photon-induced tunneling currents were successfully generated and detected by differential conductance (d_I_/d_V_) measurement of tetrathiafulvalene (TTF) and tetracyanoquinodimethane (TCNQ) molecules with STM setup upon laser pulse induction. Angstrom scale spatial resolution and sub-femtosecond temporal resolution were simultaneously accomplished, which opened a new avenue to the study of molecular electron dynamic behavior in intricate systems, with no necessity of reconstruction techniques.

## 4. Self-Assembled Structures Controlled by Voltage Induction

**Figure 3:**
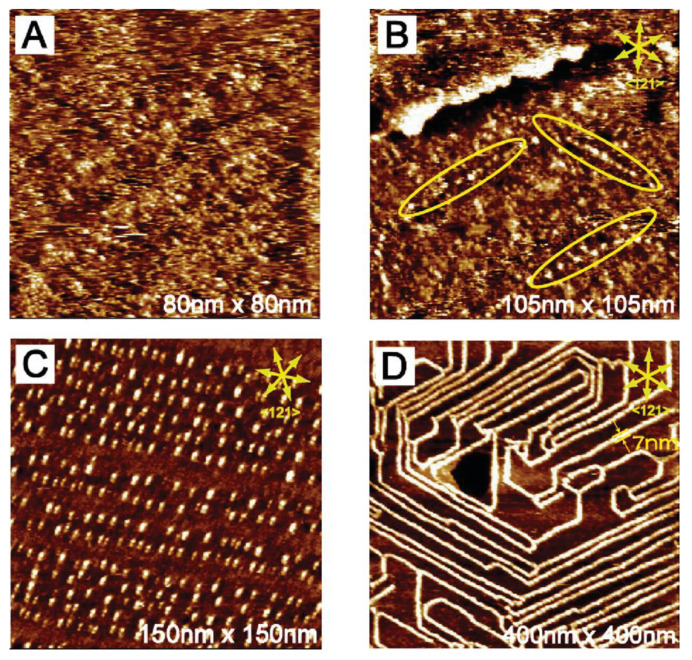
EC-STM images of picric acid on Au (111) in 0.1 M HClO_4_ with the substrate potential at (**A**) 500 mV, (**B**) 360 mV, (**C**) 250 mV, and (**D**) 200 mV. Reproduced with permission from ref. [55]. Copyright 2008, American Chemical Society.

An electric field is an efficient way to control the surface self-assembly process of molecules. The surface structural transition influenced by the electric field is indicative of the modulation of the interaction between the substrate and the molecule, as well as potential changes in intermolecular interactions, which normally lead to the order–disorder transition, surface coverage manipulation and polymorph switch. Lee et al. reported that a reversible transition between low-density porous and high-density nonporous SAM networks of 1,3,5-tris(4-carboxyphenyl)-benzene (BTB) molecules was successfully achieved by applying bias voltage with opposite signs through STM, due to the protonation or deprotonation process of the BTB molecules [53]. Based on this porous–nonporous structure transition, a controlled capturing and releasing of guest molecules was accomplished at the solution–solid interface. Additionally, the voltage induction was also feasible to initiate the phase transformation between the SAMs and covalent organic frameworks (COFs). Cai et al. formed a SAM of 1,3,5-tris(4-biphenylboronic acid)benzene (TBPBA) on HOPG with a closely packed structure under ambient conditions with a positive bias voltage applied to the substrates and the molecules [54]. When the substrate and the molecules were applied with negative bias voltage, the noncovalently bonded TBPBA molecules were arranged into a loosely packed porous structure with the co-adsorption of 1-octanoic acid solvent molecules as the guests inside the cores and more interestingly the boronic acid groups of each TBPBA molecule formed covalent bonds with the three adjacent molecules. The electric-field-induced phase transition between the SAM and COF was totally reversable independent of the polarity of the applied bias voltage. However, this method was just able to control the local structure of molecules around the STM tip. Luckily, applying an electrochemical STM (EC-STM) system, which allows to independently control the tip and substrate voltage, can globally control the phase transition process over the entire substrate. Wen et al. reported a sequential control of the polymorph change of SAM of nitrobenzene and picric acid on Au (111) substrate in 0.1 M HClO_4_, which was recorded by an EC-STM [55]. At surface potential of 550 mV, nitrobenzene molecules formed a well-ordered SAM in a ($\sqrt{3}\;$× $\sqrt{3}$) organization. The molecules started to nucleate into organic islands at 300 mV and further transformed into 1D molecular wires at 220 mV. By holding such substrate potential for 4 h, the density and continuity of the molecular wires expanded. This phase transition was caused by the irreversible reduction of the nitro groups into hydroxyamino and amine groups depending on the applied substrate potential. In contrast, picric acid formed a disordered phase at 500 mV (Figure 3A), and bright clusters started to appear at 360 mV (Figure 3B). More clusters grew by shifting the substrate potential to 250 mV and they were aligned following the preferred directions, see Figure 3C. Finally, at 200 mV, the aligned clusters were connected into molecular wires along the reconstruction lines of Au (111), see Figure 3D.

Besides the electrochemically responsive molecules, charged molecules can also be induced by the substrate voltage to form transformable SAM. Cui et al. investigated the self-assembly behavior of 9-phenylbenzo[1,2]quinolizino-[3,4,5,6-fed]phenanthridinylium perchlorate (PQPClO_4_) on Au (111) in 0.1 M HClO_4_ [56]. When the substrate potential is much more positive at 800 mV, the adlayer of PQP amorphized due to the strong repulsive force between the accumulated positive charge on the substrate and the positive charged molecules. When the substrate is less densely charged at 600 mV, an ordered porous structure was observed in the STM image and the PQP cations rotated by 90° in order to enlarge the pore size for the accommodation of an extra PQP cation as the guest into the pores at 400 mV. When the substrate voltage was negatively shifted to 150 mV, a completely different polymorph was observed in the STM image. The PQP cations were arranged into high-density linear structure with additional PQP cations forming double rows in a second layer. Other than alteration of the surface charge density, it is also possible to tune the charge density of the molecules for the voltage-induced phase transition by an EC-STM. Sagara et al. demonstrated the faradaic phase transition of SAM of dibenzyl viologen (dBV) molecules on HOPG upon a voltage-induced redox process in which the potential driven transition between dBV^•+^ and dBV^2+^ was achieved by adjusting the potential to be more negative or positive than the redox peaks [57]. The EC-STM image showed that the 2D linear pattern of the reduced form dBV^•+^ was formed at the substrate potential of −0.38 V following the three main symmetric axes of HOPG, while the oxidized form dBV^2+^ formed a gas-like adsorption layer at the potential regions that was more positive than the cathodic transition potentials due to the mobility of the molecules caused by the intermolecular electrostatic repulsion and adsorption–desorption dynamic equilibrium. Later, Huynh et al. investigated the same system on HOPG and graphene substrates in more depth by identifying three different redox states of dBV rather than the two states observed by Sagara [58]. Similarly, a mobile phase of dBV^2+^ was formed by controlling the substrate potential to the more positive regions than the redox peaks, while a condensed linear stacking phase (dBV^0^) was observed at the substrate potential that was more negative than the redox peaks. Surprisingly, a dimer phase was identified at the potential regions between the two redox peaks.

## 5. Self-Assembled Structures Regulated by Flow-Induction

The manipulation of the spatial organization of molecular components on surfaces is a crucial aspect in the bottom-up approach for the development of nanotechnologies. The supramolecular self-assembly process offers a promising method for constructing ordered patterns through the spontaneous arrangement of specifically designed molecular building blocks. However, this process involves intricate intermolecular, intramolecular, and molecule–substrate interactions, which are challenging to control. As a result, it often leads to the formation of polycrystalline or amorphous structures at the sub-micrometer scale. Recently, the shear flow method was introduced to align macromolecules and liquid crystalline polymers [59,60,61]. Inspired by this, Lee et al. introduced an applicable shear-flow method for globally guiding the self-assembly behavior of building blocks by dragging the building block containing solution with a piece of ultra-clean tissue following a certain direction to induce the long-range alignment of 2D nanopatterns of SAM of 3,6,11,14-tetradodecyloxydibenzo[g,p]-chrysene (DBC) [62]. Before shear-treatment, the DBC molecules formed a typical face-on orientation for polyaromatic molecules, while it transformed into an edge-on orientation with the molecules aligned following the [01 $\bar{1}$0] direction of the HOPG substrate. This transition can be clearly indicated by the alteration of the lattice parameters of adlayer and the spacing reduction on the neighboring molecules from 1.7 to 0.5 nm. Remarkably, this method allows the formation of DBC molecules with a singular domain up to ∼7 mm. Dragging the solution along the direction parallel to the unit cell vectors of HOPG is critical to fabricate such a large domain with a unique alignment, otherwise multiple small domains can be formed following the three main symmetric axes of HOPG due to the strong molecule–substrate interactions. Furthermore, the aligned SAM was able to maintain its arrangement by overcoming the local defects on the substrate such as step-edges. However, the existence of many defective areas within the long-range ordered SAM remained as a big issue, which determined the quality of the SAM-based molecular electronics. The authors optimized the SAM quality using a different molecular system, in which the oligomer molecules self-assembled in the solution phase into ordered 1D chains following the main symmetric axes of the substrate due to the strong molecule–substrate interactions [63]. The shear-flow treatment converted the directional multiple domains into a unidirectionally singular domain following the flow direction with ultra-low defective areas less than 0.5%, compared to that of 5% without the treatment. The spatial ordering control was further extended to the third dimension by controlling the alignment of the multilayer molecular assembly process.

**Figure 4:**
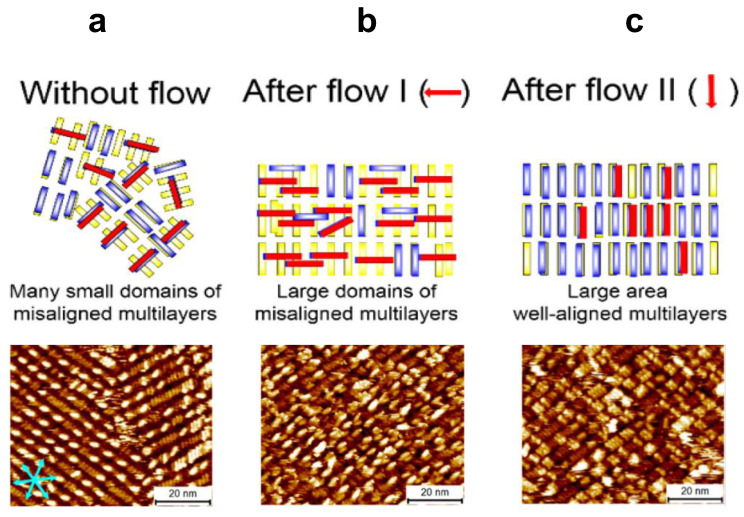
The scheme of two-step flow induced fabrication of long-range ordered HDI thin films on HOPG. (**a**) Without flow; (**b**) after flow I; (**c**) after flow II. Cyan arrows indicate graphite symmetry axes. The rectangles with different colors represent molecules adsorbed in different layers. Red arrows indicate flow direction. Reproduced with permission from ref. [64]. Copyright 2014, American Chemical Society.

Lee et al. used SAM of hexarylene diimide (HDI) as an example for demonstrating the multilayer alignment via shear flow [64]. Without any external induction, HDI formed a linear structure with many small domains on the first layer following the three main symmetric axes of HOPG and misaligned multilayers, see Figure 4a. With the first-step shear flow treatment with the flow direction perpendicular to the linear rods, the small domains on the first layer merged as a large and unidirectional domain with the molecules on the multilayer remaining misaligned, see Figure 4b. Following the second-step shear flow treatment with the flow direction parallel to the linear rods, the molecules on the multilayers were also unidirectionally aligned according to the flow directions, see Figure 4c. Surprisingly, Elemans et al. also managed to form well-ordered SAM of porphyrin trimers with an ultra-large domain size in millimeter scale, upon applying macroscopic dewetting of the solvent to create a flow force for the guidance of the porphyrin macrocycles SAM alignment [65]. This method was proven to be effective for directing the formation of large-domain liquid crystals.

## 6. Tip-Induced Self-Assembled Structures

STM is a powerful tool for the direct visualization of the structure and the lattice parameters of the SAMs at the atomic resolution. Meanwhile, its tip, which is normally used for the surface structure probing, can also be useful for the manipulation of the adlayer structure of SAMs. Initially, short-period voltage by the pulse function was applied to the adlayer molecules to tune their local electronic states through the STM tip for the induction of the structural transition of the adlayer. Alemani et al. observed the transformation between the trans- and cis-phase of 3,3′,5,5′-tetra-tert-butylazobenzene (TBA) on Au (111) substrate under ultra-high vacuum conditions [66]. At first, a thermodynamically favorable trans-isomer of TBA spontaneously adsorbed on Au (111), arranging into ordered islands with parallel rows. By applying a 2 V voltage pulse on the adlayer, the trans-isomer of TBA molecule can be converted to the cis-isomer and the precisely reverse induction can be achieved by applying another 2 V voltage pulse on the same molecule due to the voltage induced shift of molecular lowest unoccupied molecular orbitals (LOMO). Similarly, Mali et al. found a tip-pulse-induced phase transition behavior between the hexagonal motif (α phase) of SAM of PQPClO_4_ and the self–host–guest structure (β phase) with one extra PQPClO_4_ molecule located in the core formed by the α phase [67]. The energy provided through the STM tip pulsing was responsible for the transformation of SAM configurations. Recently, the STM tip has been used as a nanolithography tool to remove certain grafted defects on substrates for the selective mergence and alignment of SAM molecular domains. Bragança et al. observed the phenomena that the large domains of SAM of 5-octadecyloxy-isophthalic acid (ISA-OC18) on HOPG were interrupted into small pieces by the covalently grafted low-density diazonium molecules [68]. These grafted molecules worked as domain boundaries for the containment of the domain size of SAM. The selective Ostwald ripening process, characterized by the growth of larger domains at the expense of smaller ones, was achieved by precisely removing specific grafted molecules located on the boundaries of the domains using the STM tip. Following a similar strategy, a fully grafted substrate was nano-shaved by the STM tip to create nano-corrals for further space-confined self-assembly process. The tip-induced confinement effect has a significant impact on the self-assembly behavior of molecules, including the alignment of domains, selective chirality and phase transition of SAMs. For example, Verstraete et al. used HOPG with high-density grafting of diazonium molecules as a template, on which well-defined and stable patterns can be formed for studying the ex situ and in situ self-assembly behavior of molecules via nano-shaving with the STM tip [69]. The categorization of ex situ and in situ were defined by the absence or presence of 10,12-pentacosadiynoic acid (PCDA) solution on the substrate surface during the nano-shaving process. On a pristine HOPG (unconfined), the PCDA molecules formed a linear structure following the three main symmetric axes of HOPG with multiple domains, as expected. In an ex situ corral with 180 nm × 180 nm size, the self-assembly behavior of PCDA is similar to that on the unconfined HOPG and no alignment effect was observed. For comparison, the PCDA molecules aligned unidirectionally following their molecular long axis to form a large domain in the in situ corral.

**Figure 5:**
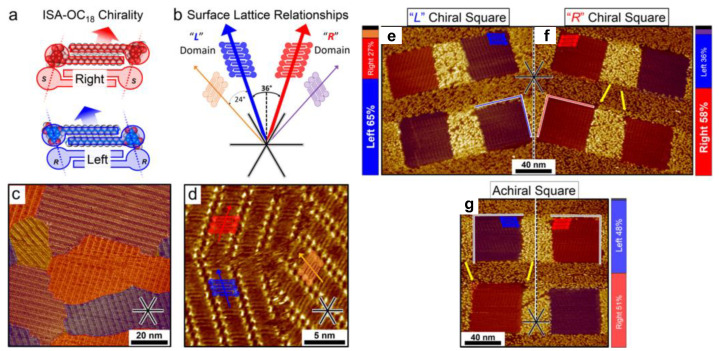
The scheme showing (**a**) ISA-OC18 S and R chirality and (**b**) L and R domain with respect to the HOPG surface lattice direction [210]; STM images of (**c**) large-area and (**d**) small-area SAM of ISA-OC18 on HOPG without confinement; STM images of (**e**) SAM of ISA-OC18 in L chiral square, (**f**) R chiral square and (**g**) achiral square. The yellow arrows point at the occasional imperfections at nanocorral borders. Reproduced with permission from ref. [70]. Copyright 2018, American Chemical Society.

Additionally, the tip-created chirality in these nano-corrals were useful to build chiral surfaces for biasing the enantiomorphic assembly of a prochiral molecule during the self-assembly process. In Figure 5, Seibel et al. demonstrated the possibility of using nanoconfined corrals with different chirality for the induction of selective adsorption of chiral polymorphs [70]. Figure 5a showed that self-assembly of prochiral 5-octadecyloxy-isophthalic acid (ISA-OC18) molecules into chiral lamellae with interdigitated alkyl-chains was observed with S (left) or R (right) chirality according to their tilt direction. These chiral lamellae preferentially adsorb on the HOPG surface to form L or R domains, which can be classified by the tilt direction with respect to the [210] surface direction of HOPG, see Figure 5b. The large-area and small-area STM images in Figure 5c and d showed an equivalent adsorption of L and R domains on the surface without any confinement. Figure 5g showed that no chiral biasing effect was observed during the self-assembly of ISA-OC18 in achiral nano-corrals based on a large number of statistical analyses. However, when the nano-corrals were created with different chirality by adjusting their tilt angles of 18° left and right with respect to the [210] surface direction of the HOPG substrate, the preferential adsorption of enantiomorph was observed in those chiral nano-corrals. In the nano-corrals designed for left-handed enantiomorphs, a majority of 65% exhibited a preference for adsorption, see Figure 5e. However, this preference decreased to 36% in the right-handed nano-corrals, see Figure 5f. In contrast, only 27% right-handed enantiomorphs tended to adsorb in the left-handed nano-corrals, which increased to 58% in the right-handed ones.

## 7. Conclusions

Self-assembly is a widely observed and significant phenomenon in the natural world. The study of adsorption and self-assembly at surfaces and interfaces is a crucial area of investigation in physical chemistry, as it is closely linked to various fundamental and practical scientific inquiries such as chirality, host–guest chemistry, and molecular electronics. In the field of nanoscience and nanotechnology, self-assembly is increasingly recognized as a prominent method in the “bottom-up” approach for constructing molecular nanostructures. The advent of STM has provided researchers with “visible” evidence in studying the self-assembly process. Gaining a comprehensive understanding of the underlying physical chemistry mechanisms involved in adsorption and self-assembly on surfaces and interfaces is a crucial initial step in the development of controllable processes. It is widely recognized that surface self-assembly is influenced by the interplay between the interactions of molecules with the substrate and with each other. The molecule–substrate interaction plays a role in determining the orientation of molecules in the adlayer, while intermolecular interactions are responsible for the formation of ordered molecular nanostructures in two dimensions. A thorough comprehension of the driving forces behind the self-assembly process enables the customization of this process. This can be achieved through external stimuli, such as temperature, electric field, photo-irradiation, shear flow, tip induction and other factors. In order to meet the requirement of manipulation of SAMs in more complex systems, combining more than one stimulus source may be necessary for programmed regulation of supramolecular structures. Additionally, there is a big gap between basic understandings and practical applications. Based on those principal comprehensions, we may devote more efforts towards the exploration of stimuli-controlled applications. For example, we may make a smooth transfer from the photo-induced tunneling currents to photoelectric devices. In addition, we may be able to push the voltage-induced SAMs phase transition to electrical devices with selectively voltage-triggered data storage, reading and erasing functions. In the realm of nanotechnology applications, surface 2D ordered structures hold significant perspectives for nanopatterning and high-density data storage. The utilization of self-assembly techniques in nanolithography offers several desirable attributes, including inexpensiveness, rapid processing, and high resolution. However, one of the primary challenges that must be addressed is the compatibility of these techniques with the existing lithography methods. Nevertheless, we maintain a positive outlook on the future of self-assembly techniques in nanofabrication applications. For example, the creation of nanoconfined spaces for further self-assembly upon the employment of the STM tip can open up possibilities for the development of innovative engineered materials with nanometer precision. The integration of lithographic and self-assembly methods can lead to the development of a diverse array of directed assembly techniques that possess enhanced capabilities beyond those of their individual components.
